# Supplementary material for: Intraperitoneally Administered Vancomycin in Patients with Peritoneal Dialysis-Associated Peritonitis: Population Pharmacokinetics and Dosing Implications
Source: Pharmaceutics. 2023 May 2;15(5):1394. doi: 10.3390/pharmaceutics15051394 (PMC10222948; doi:10.3390/pharmaceutics15051394)
Supplement: Supplementary file 1 [file pharmaceutics-15-01394-s001.zip › pharmaceutics-2360712-supplementary.pdf]

pharmaceutics

Supplementary Materials: Intraperitoneally Administered Vancomycin in Patients with Peritoneal Dialysis Associated Peritonitis: Population Pharma-cokinetics and Dosing Implications

Jan Miroslav Hartinger [1,*,#], Danica Michaličková [1,#], Eliška Dvořáčková [1], Karolína Hronová [1], Elke H.J. Krekels [2], Barbora Szonowská [3], Vladimíra Bednářová [4], Hana Benáková [5], Gabriela Kroneislová [5], Jan Závora [5], Vladimír Tesař [4] and Ondřej Slanař [1]

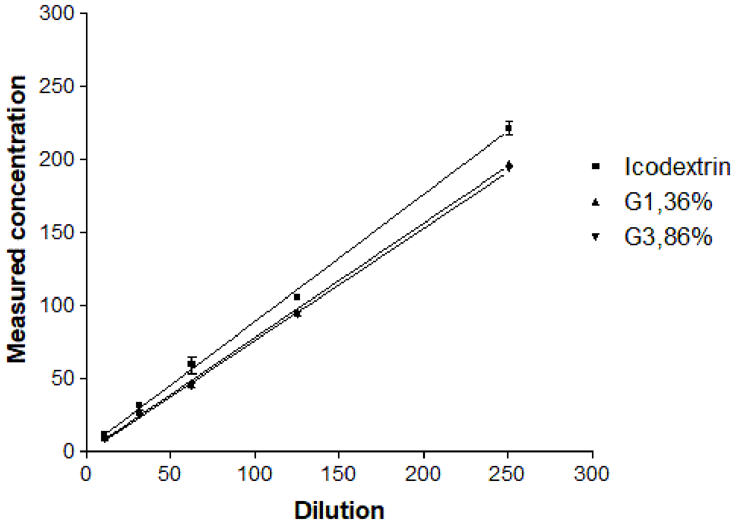

Figure S1. Calibration curves for vancomycin concentration in glucose and icodextrine solutions.

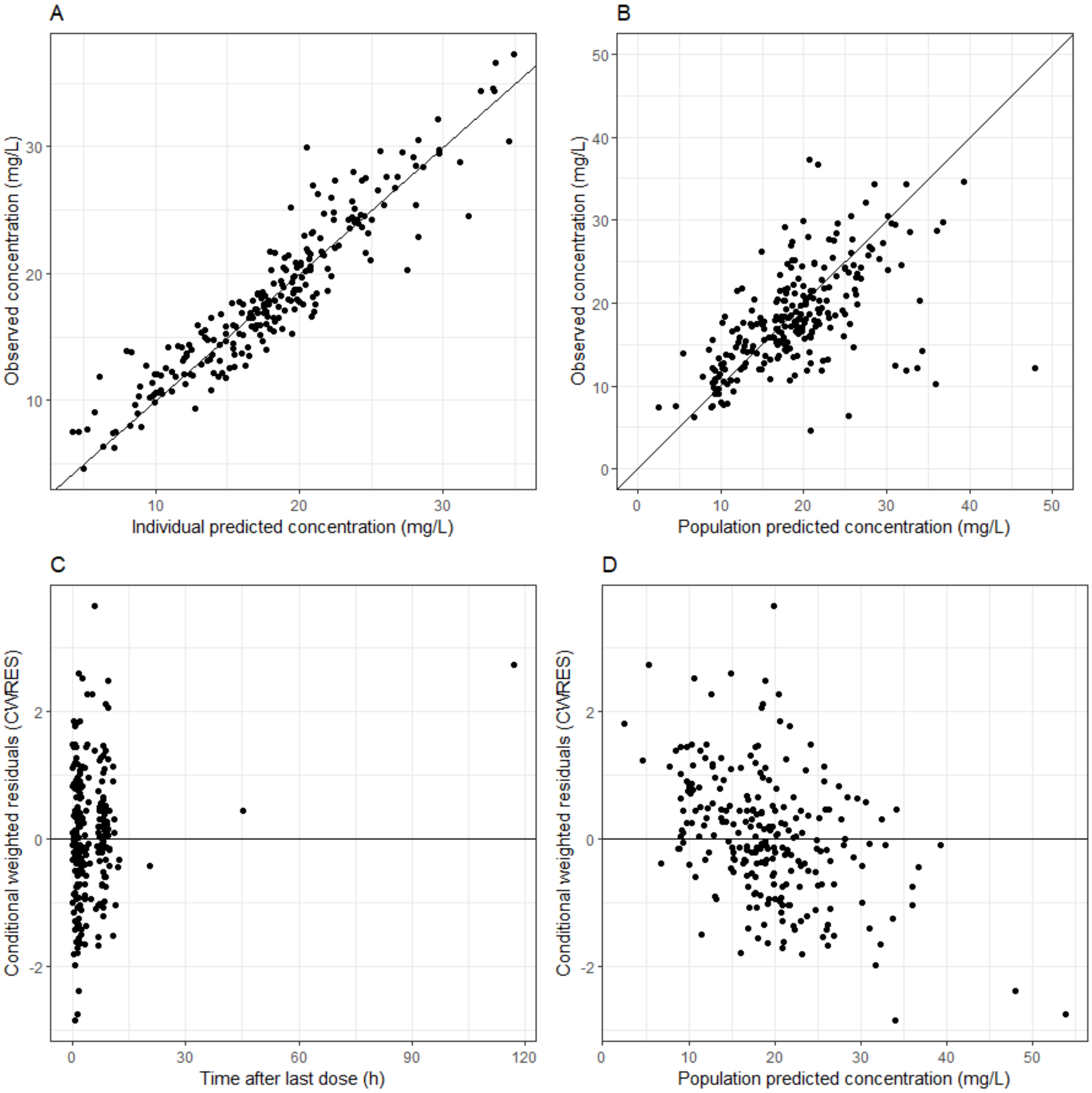

Figure S2. Goodness-of-fit plots for the final model for vancomycin concentrations in plasma in ESRD patients with peritonitis receiving CAPD. (A) Population predicted vancomycin concentrations vs. observed vancomycin concentrations. (B) Individual predicted vancomycin concentrations vs. observed vancomycin concentrations. (C) Conditional weighted residuals (CWRES) vs. population predicted vancomycin concentrations. (D) Conditional weighted residuals (CWRES) vs. time after last dose.

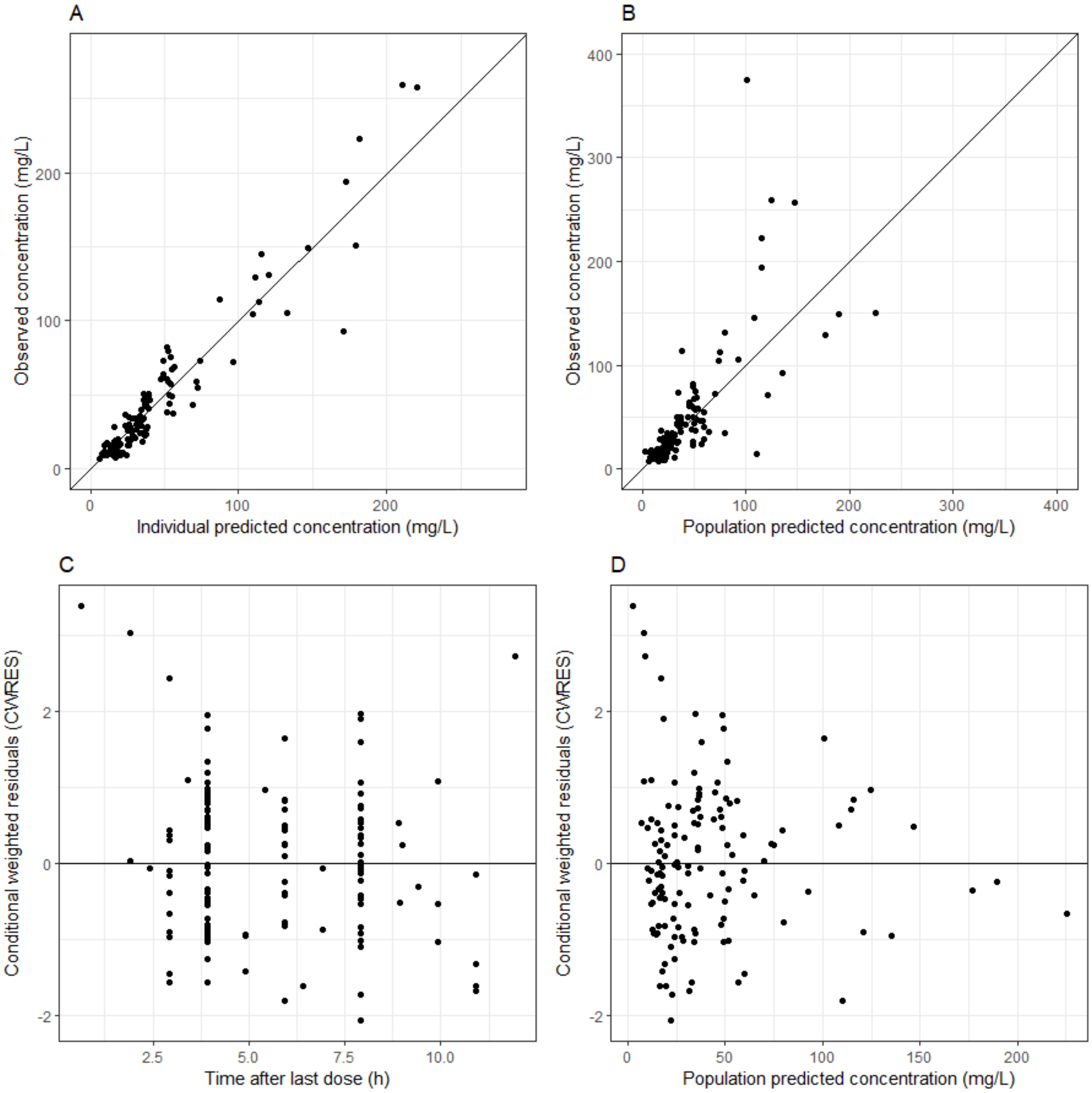

Figure S3. Goodness-of-fit plots for the final model for peritoneal vancomycin concentrations in ESRD patients with peritonitis receiving CAPD. (A) Population predicted vancomycin concentrations vs. observed vancomycin concentrations. (B) Individual predicted vancomycin concentrations vs. observed vancomycin concentrations. (C) Conditional weighted residuals (CWRES) vs. population predicted vancomycin concentrations. (D) Conditional weighted residuals (CWRES) vs. time after last dose.

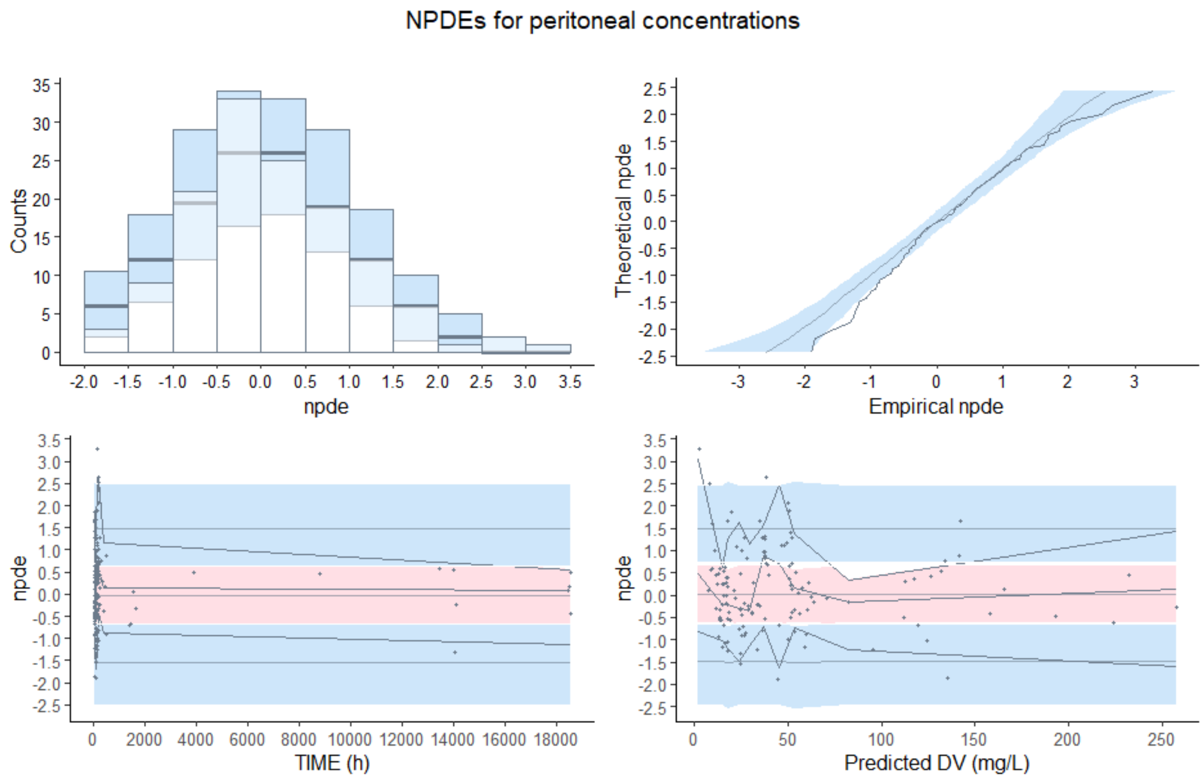

Figure S4. Normalized prediction distribution errors (NPDEs) for peritoneal vancomycin concentrations. Shown at the top are the QQ plot and histogram of the NPDEs in the overall dataset. The red dotted line and blue shaded areas show the expected trends and 95% confidence intervals of these trends, while the dark blue lines and bars show the observed NPDE distributions. At the bottom, the individual NPDE values for each observation are plotted versus time and versus the predicted concentrations with the symbols. The solid lines in the bottom graphs indicate the mean (red) and the 95% percentiles (blue) of the NPDEs, and the shaded areas are the simulated 95% confidence intervals of the NPDE median (red) and 95% percentiles (blue), while the dotted red and blue lines show the expected values for the median and 95% percentiles.

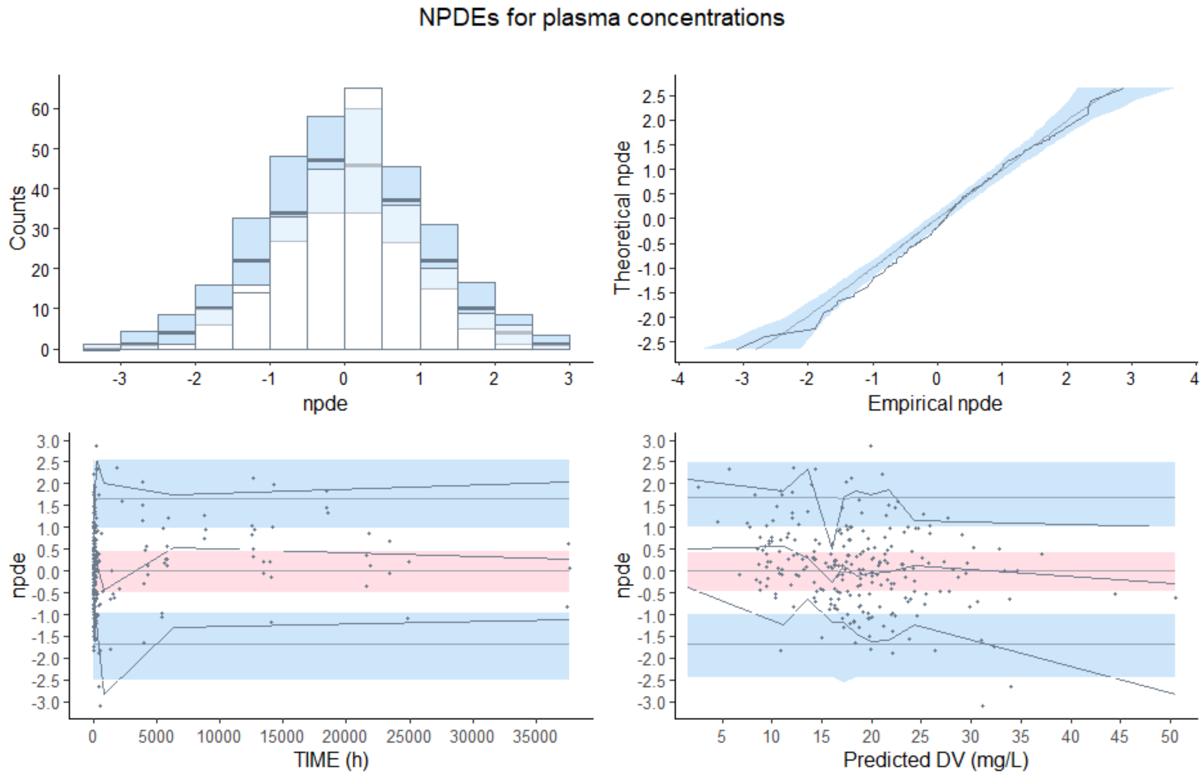

Figure S5. Normalized prediction distribution errors (NPDEs) for peritoneal vancomycin concentrations. Shown at the top are the QQ plot and histogram of the NPDEs in the overall dataset. The red dotted line and blue shaded areas show the expected trends and 95% confidence intervals of these trends, while the dark blue lines and bars show the observed NPDE distributions. At the bottom, the individual NPDE values for each observation are plotted versus time and versus the predicted concentrations with the symbols. The solid lines in the bottom graphs indicate the mean (red) and the 95% percentiles (blue) of the NPDEs, and the shaded areas are the simulated 95% confidence intervals of the NPDE median (red) and 95% percentiles (blue), while the dotted red and blue lines show the expected values for the median and 95% percentiles.

A. LD=25 mg/kg + MD=25 mg/L in each dwell, 1.5 L exchange, preserved diuresis, BW=50 kg

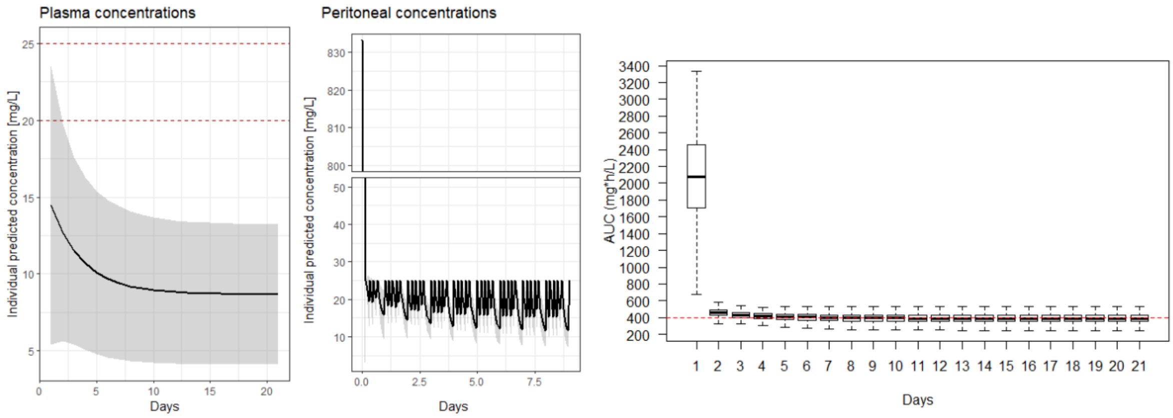

B. LD=25 mg/kg + MD=25 mg/L in each dwell, 1.5 L exchange), oliguria, BW=50 kg

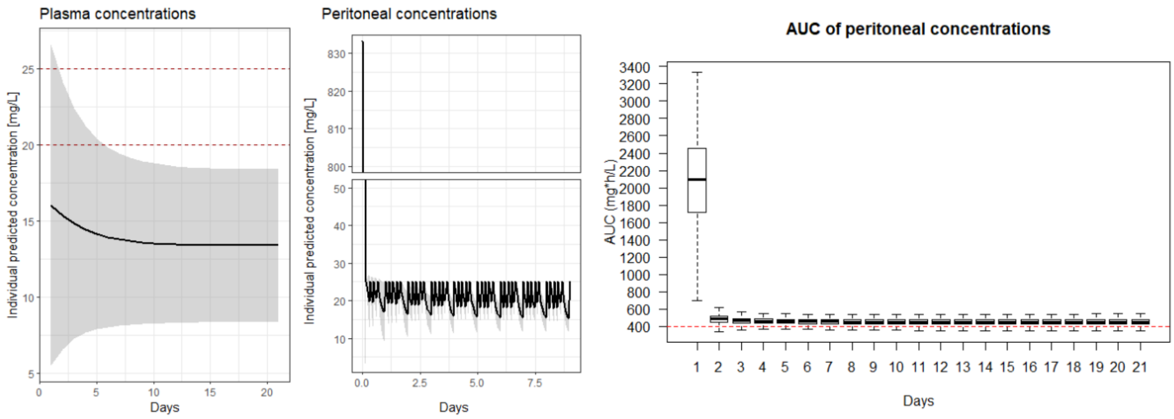

C. LD=25 mg/kg + MD=25 mg/L in each dwell, 2L exchange, preserved diuresis, BW=100 kg

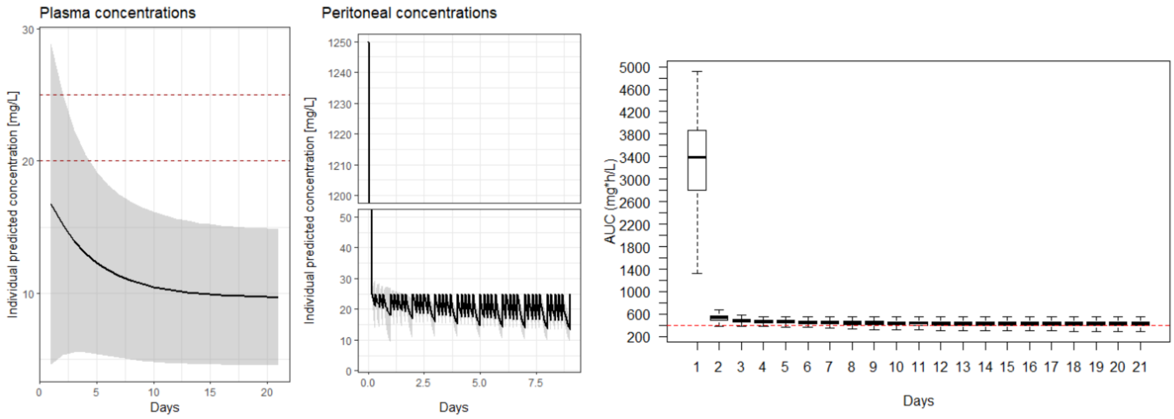

D. LD=25 mg/kg + MD=25 mg/L in each dwell, 2L exchange, oliguria, BW=100 kg

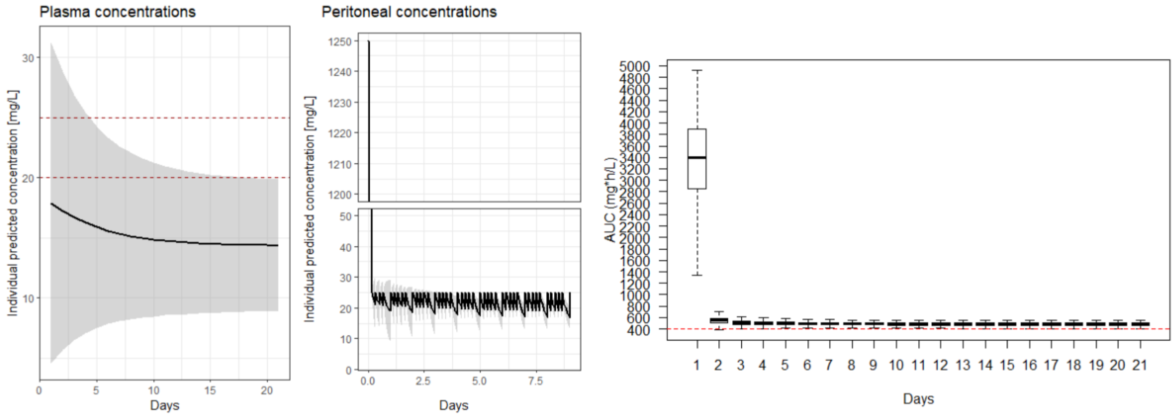

Figure S6. Simulations of vancomycin exposure in the peritoneal fluid and plasma upon recommended continuous ISPD dosing schedule. AUC_{24} is calculated for a CAPD schedule with 5 daily exchanges (i. e. 4 x 4 hours and 1 x 8 hours) in a patient with BW = 50 kg and in a patient with BW = 100 kg. When preserved diuresis is stated, residual eGFR was set to 6.76 mL/min (median value of the model). Red lines in the middle graphs – target plasma concentrations range for continuous vancomycin infusion, red line in the right graphs – target vancomycin AUC (i. e. 400 mg*h/L) that should be reached for effective therapy. AUC – area under the curve of peritoneal concentrations. LD – loading dose, MD – maintenance dose. Oliguria = less than 500 mL/day; Preserved diuresis = more than 500 mL/day. Solid lines represent median of simulated concentrations, shaded areas represent 95% confidence interval of simulated concentrations; box plot with whiskers from minimum to maximum are presented for AUC_{24} for 21 day-long treatment.

A. LD=20 mg/kg + MD=50 mg/L in each dwell, 2L exchange, preserved diuresis, BW=50 kg

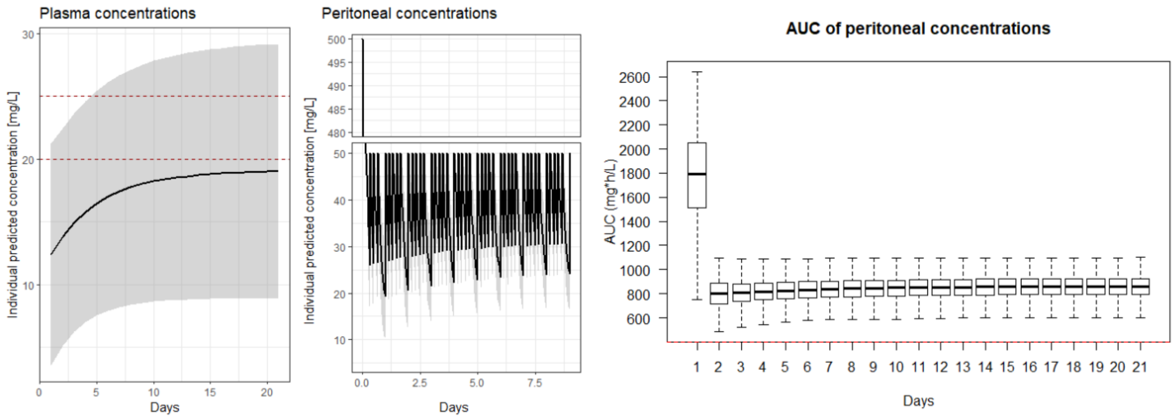

B. LD=20 mg/kg + MD=50 mg/L in each dwell, 2L exchange, oliguria, BW=50 kg

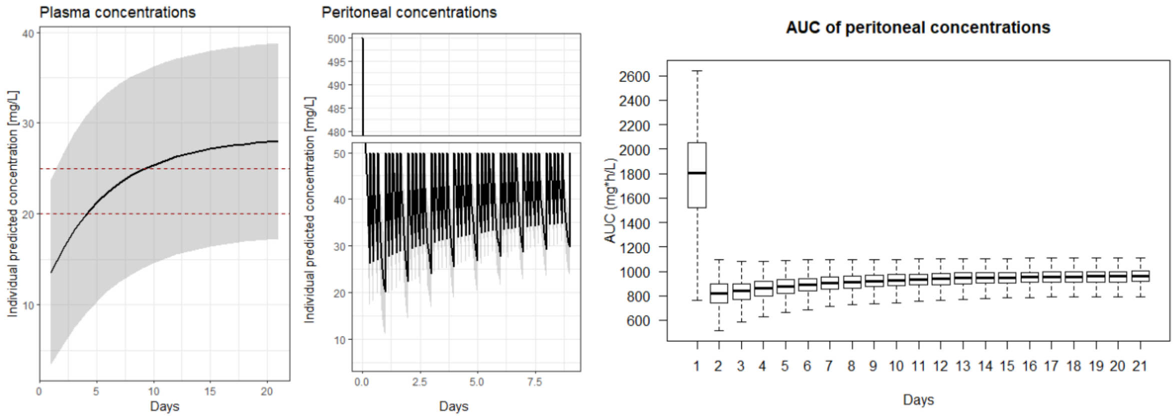

Figure S7. Expected vancomycin i. p. and plasmatic concentrations and exposure after proposed dosing. AUC_{24} is calculated according for a CAPD schedule with 5 daily exchanges (i. e. 4 x 4 hours and 1 x 8 hours) in patients with BW = 50 kg and 2L exchanges. When preserved diuresis is stated, residual eGFR was set to 6.76 mL/min (median value of the model). Red lines in the middle graphs – target plasma concentrations range for continuous vancomycin infusion, red line in the right graphs – target vancomycin AUC (i. e. 400 mg*h/L) that should be reached for effective therapy. AUC – area under the curve of peritoneal concentrations. LD – loading dose, MD – maintenance dose. Oliguria = less than 500 mL/day; Preserved diuresis = more than 500 mL/day. Solid lines represent median of simulated concentrations, shaded areas represent 95% confidence interval of simulated concentrations; box plot with whiskers from minimum to maximum are presented for AUC_{24} for 21 day-long treatment.
